# Successful Method of Filling Fast Decaying Teeth of the Young and Anemic

**Published:** 1898-01

**Authors:** C. Bunting Colson

**Affiliations:** Charleston, S. C.


ARTICLE VIII.

### SUCCESSFUL METHOD OF FILLING FAST DECAYING TEETH OF THE YOUNG AND ANEMIC.

BY C. BUNTING COLSON, D. D. S., CHARLESTON, S. C.

Read before White Mountain Meeting, July, 1897.

It is my belief that we are on the eve of discovering a specific which will prevent disintegration of the teeth of the young and of those in poor health, other than by the merely mechanical and prophylactic measures of to-day. Before explaining my own methods of procedure, I will discuss briefly some of the processes and materials which have given us particularly satisfactory results in the the mouths of the young and anemic, and especially of nursing moth rs. The most useful materials, here named in the order of their usefulness, have been soft gold, guttapercha, tin, lead, cohesive gold, oxyphosphate of zinc and oxychlorid of zinc. The various processes upon which we have depended have been fillings with one or more of the above materials; cleansing or polishing of the enamel; or completely surrounding the teeth with metal as with a band or crown, thus protecting them from the secretions of the mouth. Therapeutically we have advocated prophylaxis by friction with the brush, silk, mouth lotions and dentifrices, and cauterizing with nitrate of silver of surfaces preventing superficial caries.

In which direction shall the dental student of the future seek for that specific which shall be to dentistry what Jenner, Pasteur, Koch, Roux and others have given to the general practitioners of medicine? Preventive dentistry may eventually rely upon a process, certainly not upon any particular filling material. It might be possible to permanently preserve human teeth if a method could be discovered by which we could electrically deposit gold, silver, nickel or platinum over all the surface of the teeth of the young, preventing by such deposit caries in fissures, and the attacks of micro-organisms; but as we can scarcely hope for this, and as filling materials can only retard and partially prevent further progress of rapid decay, and as such disintegration is pathological, our future dependence must be upon therapeusis.

It has long been noted that pure soft gold apparently acts better than any other material used in preventing subsequent disintegration of the teeth filled, even where the manipulation has been faulty. It is not uncommon to find a leaking or loose gold filling of soft gold remaining in a tooth for years and preserving it, which would seem to prove that its action depends upon some therapeutic property and is not due to perfect adaptation. This can not be said of cohesive gold.

We have similar preservative properties in tin and lead, but it is not always wise to use these materials in the teeth of the young because of the discoloration which they frequently cause. Copper amalgam has given good results under certain conditions, of which, however, as yet we know too little to be able to utilize this material, which the lack of knowledge as to when and where to use it renders most unreliable.

We have fairly good results from gutta-percha as a temporary expedient, and here again we have an action which is apparently therapeutical rather than mechanical. When well manipulated, gutta-percha often serves a permanent purpose, and even when poorly placed frequently gives better results than could be obtained with any other material.

Therapeutics has furnished us with some good preparations for the prophylaxis of the mouth, but none are specific, and the mouth being an ever washed cavity, the effect of the most powerful agent is soon lost. In many mouths the teeth are covered with a viscid, thick, clinging deposit, not very soluble and not easily removed, and which is not penetrated by any ordinary or even extraordinary germicide or antiseptic. Wash a child's mouth with the most potent of these agents yet known, and we still find under the felt-like pads an abundance of thread-like bacteria.

We have several mechanical processes which are valuable in combating the early stages of decay. When first detected, if we thoroughly cleanse the surface and polish the enamel with hardwood polishers, we are frequently able to abort the process of decay. We also have a reliable agent in nitrate of silver, especially for deciduous teeth, where it is of great advantage in retarding the progress of disintegration of the enamel, and, except that it leaves a coal black stain, it would be exceedingly valuable in dentistry.

I will now briefly describe my method of preserving rapidly decaying teeth in the mouths of patients between the ages of ten and twenty, and also in young mothers and anemics. We find similar conditions in nearly all these cases, except that in children we have a larger pulp cavity and less dentin between filling and pulp. Many years ago I became impressed with the idea that the enamel and dentin may be affected far beyond the point usually reached with our excavators. I was forced to this conclusion by observing the recurrence of decay around and beneath fillings. I have frequently cut away more tooth substance than is usual and obtained success, though I have occasionally lost a pulp from the proximity of the filling to that organ, even when care had been taken to line the cavity. I have also found that in many of these cases in the course of time the vitality of the pulps became impaired, the pulps eventually dying. I therefore became convinced that it was not altogether the proximity of the cement lining nor of the gold which caused this, but rather the infected condition of the dentin between the filling and the pulp, and I have proven the correctness of this theory by obtaining good results through proper treatment prior to filling. Having satisfied myself that the dentin of decayed teeth is affected more deeply than is usually believed, I became assured that soft gold would be the best material for preserving the teeth and preventing subsequent recurrences of decay.

My present method of filling rapidly decaying teeth is as follows: I demand sufficient space in which to work. If the cavities are in approximal surfaces, I open by packing cotton, cork, or sometimes rubber, and then keep the teeth apart with a peg of wood until the soreness leaves. With the rubber dam adjusted and the cavity well dried, I attack the enamel edges with a very sharp chisel or excavator, but prefer to use a small, fine cut fissure drill or bur. I cut back the enamel well before I proceed to excavate the cavity. I excavate the dentin with sharp excavators, using only hand instruments, as they are better controlled, and less painful if sharp. After as thorough an excavation as judgment will permit, we are frequently compelled to leave a small pad of decay at the bottom of the cavity for fear of exposing the pulp. Believing that the apparently normal dentin and enamel are not so healthy as they seem, of course it is not satisfactory to leave this pad of decay, which is composed of particles of broken down dentin, micro-organisms and other septic material absorbed from the saliva; nevertheless, we are forced to do so. To fill over it with any material without first taking necessary precautions would be fatal to the success of the permanent filling. It is the bacteria thus left within the cavity which destroy many teeth, however carefully lined and filled.

After excavating, my next step is to dry out the cavity with warm air—and I frequently do this while excavating as it reduces pain to a minimum—which should be a slow stream of air, not hot, but just warm enough to drive back the serum and evaporate the contents of the tubuli to some extent. I then bathe the cavity with a drop of a caustic soda solution which is instantly sucked up by the dry tubuli. The soda penetrates the tissues of the tooth to a considerable depth, neutralizing all the acid that may be present in the tooth itself or in the pad of decay. Again drying this out and applying warm air as before, I flood the cavity with oil of cloves. Why oil of cloves? I have tried nearly all the germicides that I felt justified in experimenting with, and I find that oil of cloves is the most suitable and efficient. After absorbing out the surplus with bibulous paper I again apply warm air. Oil of cloves has the peculiar property, when heated to the point of volatility, of penetrating; and will pass deeply into the tubuli and so embalm the substance of the pad that no fermentative action can occur.

Now I return to the enamel border. Treatment of this margin is the most important of the entire process of filling this class of teeth. I employ some original instruments made of carborundum pencils ground down and pointed so that they resemble a well-sharpened lead pencil. I have these of fine grit and placed in porte polishers. With these fine points I grind my enamel well around to the full extent of the diameter of the cavity, so that there shall be no overhang to any portion near the surface. I now trim the extreme outer edge of the enamel, surrounding the cavity to the outer bevel or counter-sink, similar to that which is made in flushing a screw head in cabinet work, but not at so acute an angle. All this is done with the small carborundum pencil points. Then I line cavity with balsam, believing that I seal within the tubuli sufficient essential oil to prevent the development of any fermentative decomposition in the pad. While the balsam is drying I polish my beveled enamel border.

I desire to make a few remarks on polishing enamel, because the practice does not seem to be so generally appreciated as it was twenty years ago; It was formerly the common practice in the treatment of superficial decay, after removing the roughened surface, to polish the enamel carefully, and I have always been impressed with the ability of these fresh surfaces to resist the attacks of the agents of decay, which seem to prefer a roughened enamel surface. When we find decay to be general throughout a mouth, we invariably may note that the enamel surfaces are far from having the high polish which we see in a healthy mouth, therefore, I think it very essential to perfectly polish these enamel borders. I use for this purpose points of hardwood, mostly seasoned hickory and boxwood sharpened like pencils, held in the porte polisher bits. Running my engine at a rapid speed, I polish the beveled enamel borders of my cavity until my mouth mirror shows me that they shine and reflect light. This is not difficult nor painful, nor does it require much time, but it is very necessary to a perfect operation.

Bear in mind that I am writing of teeth in which we may expect a recurrence of decay. In such cases I ordinarily use a cement lining, especially in very young mouths, to avoid the necessity of placing gold in contact with the dentin, but this must be done with judgment and depends upon the depth of the cavity. If I use cement I anchor in it several pellets of cohesive gold as a foundation, in preference to deep anchorages, but I do not fill the entire cavity with cohesive gold. When I have the cavity about two-thirds filled I place around, as I work, little pellets of pure soft gold about No. 4, folded from four to six times. These I lay along the enamel borders, and they are held in place by the cohesive gold as it is built over them until I have all the enamel edges of the cavity overlaid by these soft gold pads beyond the bevel. I then complete the filling with cohesive gold and burnish thoroughly.

It may be asked, why not fill with soft gold entirely? The answer is that soft gold fillings to be well inserted require much pressure and malleting. The enamel of the teeth of the young is not dense enough to resist such pressure. Again, I cannot finish to a certainty to the outer edge of the bevel of the enamel with soft gold, and I also wish to restore the approximal relations of the teeth by contouring. Nevertheless, I want soft gold on the beveled polished edges, covered by cohesive gold well burnished down, and I have no fear of bacteria destroying the tooth so polished. A few children may not have the patience or endurance to submit to this method, though rarely am I obliged to adopt any other.

My next favorite filling is gutta-percha, and I prefer the common pink base plate gutta-percha to any of the white preparations, and I use it whenever I can, where its color does not make it objectionable. To fill teeth with gutta-percha, especially in the mouths of small children, is an art and requires as expert a hand as any gold filling, and, although it can be done much more quickly, is difficult. In the posterior teeth I always use it below the gum margin and almost invariably touch the bottom of the cavity, be it enamel, cementum or dentin, with a crystal of silver nitrate, and then insert the gutta-percha. In this manner I have saved teeth which, without the use of the silver nitrate, could not have been certainly preserved.—*Items of Interest.*

